# Inhibition of 11β-hydroxysteroid dehydrogenase 1 relieves fibrosis through depolarizing of hepatic stellate cell in NASH

**DOI:** 10.1038/s41419-022-05452-x

**Published:** 2022-11-29

**Authors:** Su-Yeon Lee, Sanghwa Kim, Inhee Choi, Yeonhwa Song, Namjeong Kim, Hyung Chul Ryu, Jee Woong Lim, Hyo Jin Kang, Jason Kim, Haeng Ran Seo

**Affiliations:** 1 Advanced Biomedical Research Lab, Institut Pasteur Korea, Seongnam-si, Gyeonggi-do Republic of Korea; 2 Medicinal Chemistry, Institut Pasteur Korea, Seongnam-si, Gyeonggi-do Republic of Korea; 3 R&D center, J2H Biotech Inc., Suwon-si, Gyeonggi-do Republic of Korea

**Keywords:** Mechanisms of disease, Target identification

## Abstract

11β-hydroxysteroid dehydrogenase type 1 (11βHSD1) is a key enzyme that catalyzes the intracellular conversion of cortisone to physiologically active cortisol. Although 11βHSD1 has been implicated in numerous metabolic syndromes, such as obesity and diabetes, the functional roles of 11βHSD1 during progression of nonalcoholic steatohepatitis (NASH) and consequent fibrosis have not been fully elucidated. We found that pharmacological and genetic inhibition of 11βHSD1 resulted in reprogramming of hepatic stellate cell (HSC) activation via inhibition of p-SMAD3, α-SMA, Snail, and Col1A1 in a fibrotic environment and in multicellular hepatic spheroids (MCHSs). We also determined that 11βHSD1 contributes to the maintenance of NF-κB signaling through modulation of TNF, TLR7, ITGB3, and TWIST, as well as regulating PPARα signaling and extracellular matrix accumulation in activated HSCs during advanced fibrogenesis in MCHSs. Of great interest, the 11βHSD1 inhibitor J2H-1702 significantly attenuated hepatic lipid accumulation and ameliorated liver fibrosis in diet- and toxicity-induced NASH mouse models. Together, our data indicate that J2H-1702 is a promising new clinical candidate for the treatment of NASH.

## Introduction

Nonalcoholic steatohepatitis (NASH) is characterized by liver inflammation and damage caused by accumulation of fat in the liver and can progress to more serious disease stages, such as advanced liver fibrosis, cirrhosis, and liver cancer. For example, NASH patients with obesity or diabetes present with advanced fibrosis upon liver biopsy [1]. Although vitamin E, pioglitazone, and liraglutide have improved liver histology in randomized trials, no Food and Drug Administration-approved drugs for NASH are available [2].

In particular, fibrosis, which is present in over 80% of NASH patients, appears to be the most important factor that affects survival [3, 4], although anti-fibrotic drugs can improve long-term outcomes. However, there remains a significant unmet need for safe and effective medications for treating hepatic fibrosis in NASH. Hepatic fibrosis results from a pattern of severe inflammation induced by the epithelial-to-mesenchymal transition (EMT), endothelial-to-mesenchymal transition (EndMT), and excessive accumulation of extracellular matrix (ECM) proteins in the liver. We previously developed a multicellular hepatic spheroid (MCHS) model using hepatocytes, fibroblasts, hepatic stellate cells, and liver endothelial cells that accurately mimics the behavior of the EMT, EndMT, and liver fibrosis heterogeneity in vivo. These spheroids are designed to gauge effectiveness of novel compounds for liver-related diseases, such as liver fibrosis [5] and hepatocellular carcinomas (HCCs) [6].

In this study, we generated advanced MCHSs by augmenting macrophages, critical regulators of fibrogenesis, with existing MCHSs. Activation of hepatic stellate cells (HSCs) in the MCHSs, which induce increased expression of α-smooth muscle actin (α-SMA) and ECM production, as well as spheroid morphological changes, is also a well-established key driver of fibrosis [7–9], highlighting the critical need to develop new strategies for reversing HSC activation and effectively treating fibrosis.

11β-hydroxysteroid dehydrogenase 1 (11βHSD1) plays an important role in regulating glucocorticoid levels (cortisol in males) and is highly expressed in the human liver [10]. Glucocorticoid excess results in impairments of the central nervous system, cardiovascular function, immune function, musculoskeletal system, and metabolic homeostasis. In particular, elevated levels of active glucocorticoid in the liver lead to insulin resistance, type 2 diabetes, hepatic steatosis, and dyslipidemia, among other conditions [11]. Although 11βHSD1 contributes to liver fibrosis via increasing active glucocorticoid, its role in liver fibrosis is still controversial [12–14]. In a previous study, we developed pyridineamide derivatives (J2H-1702, 2-((R)-4-(2-Fluoro-4-(methylsulfonyl)phenyl)-2-methylpiperazin-1-yl)-N-((1 R,2 s,3 S,5 S,7 S)-5- hydroxyadamantan-2-yl) pyrimidine-4-carboxamide) as 11βHSD1 inhibitors to treat diabetes and metabolic disease and found that J2H-1702 sufficiently inhibited hepatic gluconeogenesis and partial improved lipid profiles and metabolic parameters [15, 16].

Here, we investigated the effects of 11βHSD1 inhibition on liver fibrogenesis progression. Based on our observations, we sought to define new indications of J2H-1702 in treating liver-related diseases using newly generated MCHSs in based-high-throughput drug screening and for utilizing J2H-1702 in dietary- and toxicity-induced NASH mouse models.

## Results

### J2H-1702, an inhibitor of 11βHSD1, alleviated fibrosis in MCHSs

Four basic cell types reside in the liver. Hepatocytes are specialized parenchymal cells, and non-parenchymal cell types are principally liver sinusoidal endothelial cells (ECs), Kupffer cells, and hepatic stellate cells (HSCs). To recapitulate the important features of liver tissues, we developed advanced MCHSs, which comprise hepatocytes, HSCs, ECs, and macrophages for high-throughput screening of potential liver fibrosis inhibitors.

**Fig. 1:**
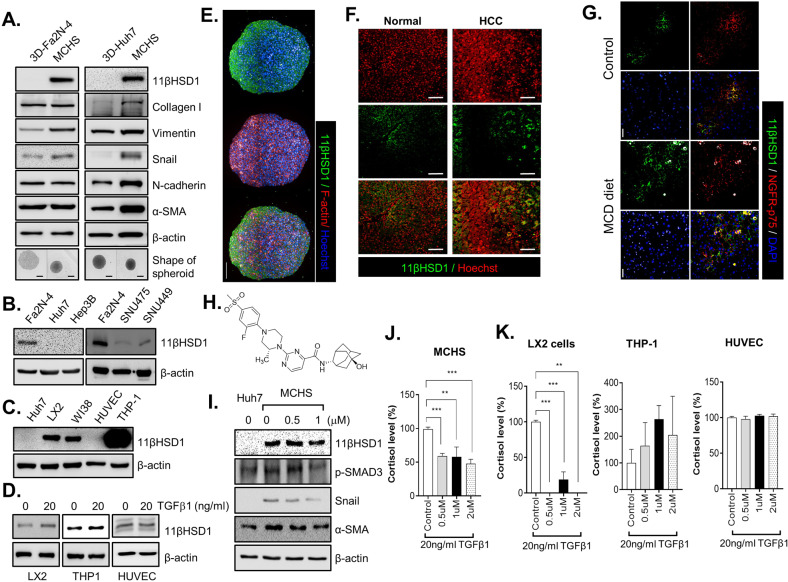
The 11βHSD1 inhibitor J2H-1702 ameliorates fibrosis in MCHSs. **A** 3D spheroids and MCHSs were formed using Fa2N-4 or Huh7 cells, respectively. Bright-field images were obtained with a 10× objective. **B**, **C** Expression of 11βHSD1 in monolayer (2D) cultures with various cell lines (Fa2N4, LX2, WI38, HUVEC, THP-1, Huh7, Hep3B, SNU475, SNU449) was detected by Western blot analysis. **D** 20 ng/ml TGF-β1 was added to LX2 cells, THP-1 cells, and HUVECs, and expression of 11βHSD1 was detected by Western blot. **E** MCHSs were co-stained with α-11βHSD and Hoechst 33342 (upper), F-actin and Hoechst 33342 (middle), or α-11βHSD1, F-actin and Hoechst 33342 (lower) to confirm expression patterns of 11βHSD in spheroids. **F** Immunofluorescence (IF) staining of 11βHSD1 in normal or tumor tissues from patients with liver cancer. **G** IHC staining of 11βHSD1 and NGFR-p75 in normal chow diet control and MCD-diet NASH mouse liver. **H** The chemical structure of J2H-1702 (11βHSD1 inhibitor). **I** Expression of 11βHSD1, p-SMAD3 (Ser423/425), Snail, and α-SMA in Huh7 spheroids alone and in 0, 0.5, or 1 µM J2H-1702-treated MCHSs was detected by Western blot. **J**, **K** Cortisol levels were measured in various cell types, including MCHS, LX2, and THP-1 cells and HUVECs co-treated with 20 ng/ml TGF-β1 and 0, 0.5, 1, or 2 µM J2H-1702. Data are shown as the mean ± SD (*n* = 3). ^**^P < 0.01 and ^***^P < 0.001 compared to the control group. Scale bar: 20 μm.

In the MCHS models, genes involved in the production of ECM structural constituents, as well as of 11βHSD1, were significantly enriched. In particular, we found that neither hepatocyte nor HCC spheroids expressed 11βHSD1; rather, its expression was only found in MCHSs (Fig. 1A). Therefore, the differential expression of 11βHSD1 in spheroids with parenchymal cells and in MCHS models may be due to interactions between parenchymal cells and non-parenchymal cells. This finding stresses the importance of MCHS models, which mimic the actual fibrotic microenvironment and thus could exhibit different protein expression patterns of 11βHSD1.

Although 11βHSD1 is more highly expressed in liver tissues relative to other tissue types, HCC cells lines (Huh7, Hep3B, SNU475, SUN449) displayed significantly lower 11βHSD1 expression than Fa2N-4 cells, a well-known normal hepatocyte cell line (Fig. 1B). These results showed that severe liver injury and fibrosis during tumorigenesis induced loss of 11βHSD1 expression in hepatocytes.

Because MCHSs are composed of hepatocytes and stromal cells, such as fibroblasts (WI38), HSCs (LX2), ECs (HUVECs), and macrophages (PMA-treated THP-1 cells) seeded at constant ratios, we investigated which cells within the MCHSs predominantly expressed 11βHSD1. 11βHSD1 was rarely expressed in Huh7 cells and HUVECs, whereas LX2 and WI38 had high expression of 11βHSD1. Notably, PMA-treated THP-1 cells showed the highest 11βHSD1 expression among all MCHS cells (Fig. 1C). Liver tissues with severe fibrosis produce TGF-β1, which activates non-parenchymal cells such as HSCs and ECs. Similarly, treatment of TGF-β1 induced increasing levels of α-11βHSD1 expression in LX2 cells, but not in other types of stromal cells (Fig. 1D).

The results of the immunohistological staining showed that the expression patterns of 11βHSD1 was compartmentalized in MCHSs (Fig. 1E). We also analyzed 11βHSD1 expression in HCC tissues and paired para-tumor liver tissues from patients using immunohistochemistry (IHC) and observed 11βHSD1 expression in a specific regions of these specimens (Fig. 1F). To confirm co-localization with 11βHSD1 and stellate cells, we performed IHC staining of 11βHSD1 and NGFR-p75 (LX2 stellate cell marker) in normal chow diet control and MCD-diet NASH mouse liver. We observed co-localization of 11βHSD1 with LX2 stellate cells (Fig. 1G).

To elucidate the functional roles of 11βHSD1 in liver fibrosis, we applied J2H-1702, a novel 11βHSD1 inhibitor, to advanced MCHSs. The structure of J2H-1702, which was generated in PubChem, is shown in Fig. 1H. Treatment with J2H-1702 inhibited the expression of α-SMA, Snail, p-SMAD3, and 11βHSD1 with a concomitant decrease of cortisol levels in MCHSs (Fig. 1I, J). Interestingly, J2H-1702 specifically inhibited cortisol production in HSCs, but not in macrophages and ECs (Fig. 1K). These results show that 11βHSD1 exerts cell type-specific effects on fibrotic liver microenvironments and prompted us to next focus on the functional roles of 11βHSD1 during activation of HSCs.

### Suppression of 11βHSD1 effectively reversed activation of HSCs

**Fig. 2:**
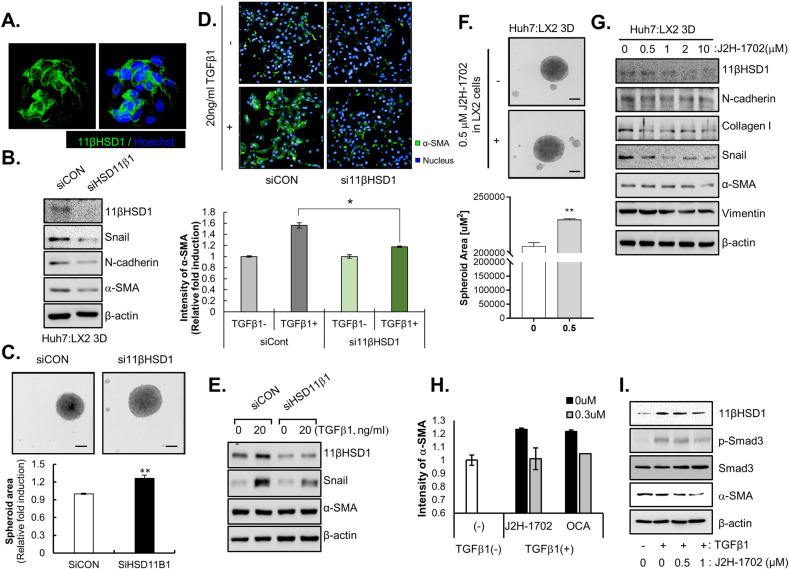
11βHSD1 knockdown reversed activation of HSCs. **A** Immunofluorescence images of 11βHSD1 expression in LX2 cells. **B** Expression of 11βHSD1 and EMT markers (Snail, N-cadherin, α-SMA) in spheroids co-cultured with Huh7 and non-specific siRNA (siCon) or 11βHSD1 siRNA (si11βHSD1)-transfected LX2 cells at a 1:1 ratio was measured by Western blot. **C** Bright-field images were captured of spheroids comprising Huh7 and control (siCon) or 11βHSD1-deficient (si11βHSD1) LX2 cells. The images were obtained using the Operetta^®^ High Content Screening System, and the spheroid area was analyzed using Harmony software. **D** Immunofluorescence images of α-SMA expression in control (siCon) or 11βHSD1-deficient (si11βHSD1) LX2 cells with or without 20 ng/ml TGF-β1 for 48 h. **E** Expression of 11βHSD1, Snail, and α-SMA in control (siCon) or 11βHSD1-deficient (si11βHSD1) LX2 cells with or without 20 ng/ml TGFβ1 for 48 h was measured by Western blot. **F** Bright-field image of a 3D spheroid composed of Huh7 and 0.5 µM J2H-1702-treated LX2 cells. **G** Expression of 11βHSD1, N-cadherin, Col1A1, Snail, α-SMA, and Vimentin in a spheroid composed of 0, 0.5, 1, 2, or 10 µM J2H-1702-treated LX2 and Huh7 cells at a ratio of 7:3 was measured by Western blot. **H** The immunofluorescence intensity of α-SMA was measured in 0 or 0.3 µM J2H-1702- or obeticholic acid (OCA)-treated cells co-incubated with 20 ng/ml TGF-β1 for 48 h and was compared to the control (TGFβ-1(-)). **I** Protein expression of 11βHSD1, p-SMAD3 (Ser423/425), SMAD, and α-SMA were detected in LX2 cells treated with 0, 0.5, or 1 µM J2H-1702 and 20 ng/ml TGF-β1 compared with the control. Scale bar: 20 μm.

In our previous study, we showed that the interaction between HSCs and HCC cells facilitates the compactness of tumor spheroids [5, 6, 17]. Because 11βHSD1 expression was elevated by the interaction between HCC cells and stromal cells in MCHSs, we examined whether the compactness of MCHSs composed of Huh7 and LX2 cells changed in response to loss of 11βHSD1 expression. 11βHSD1 mainly localized to the cytosol of HSCs (Fig. 2A). We confirmed that siRNA of 11βHSD1 (si11βHSD1) efficiently depleted 11βHSD1 protein expression in LX2 cells, whereas control siRNA (siCon) treatment did not. MCHSs with Huh7 and 11βHSD1-deficient LX2 cells displayed reduced expression of EMT-related proteins, such as Snail, N-cadherin, and α-SMA, relative to MCHSs with Huh7 and normal LX2 cells (Fig. 2B).

We next explored the shape of MCHSs with Huh7 and 11βHSD1-deficient LX2 cells via transfection of si11βHSD1 into LX2 cells to investigate the effect of 11βHSD1 inhibition on MCHS compactness. Although MCHSs with Huh7 and normal LX2 cells exhibited enhanced spheroid compactness, MCHSs with Huh7 and 11βHSD1-deficient LX2 cells did not (Fig. 2C).

To elucidate the cellular role of 11βHSD1 in the activation of HSCs during the development of liver fibrosis, we observed the morphological changes of HSCs induced by inhibition of 11βHSD1 with si11βHSD1 after TGF-β1 treatment. Control LX2 cells had a polygonal shape with low intensity of α-SMA. Treatment of TGF-β1 transformed the cells into a spindle shape and increased their intensity of α-SMA, whereas treatment with si11βHSD1 reduced TGF-β1-induced protrusions and α-SMA intensity (Fig. 2D). Inhibition of 11βHSD1 expression also decreased expression of Snail in TGFβ1-treated LX2 cells (Fig. 2E).

Next, we investigated the effect of J2H-1702 on the compactness of MCHSs composed of Huh7 and LX2 cells. Their compactness was lost by treating the LX2 cells with 0.5 μM J2H-1702 (Fig. 2F). Similarly, MCHSs with Huh7 and J2H-1702 treated-LX2 cells showed decreased expression of Snail, N-cadherin, Collagen I, α-SMA, Vimentin, and 11βHSD1 (Fig. 2G). We next measured the effects of J2H-1702 on TGF-β1-induced HSC activation using cellular phenotype-based assays. J2H-1702 inhibited the expression of α -SMA (−17.8%) after treatment with 20 ng/ml TGF-β1 in LX2 cells, with an efficacy comparable to that of 0.3 μM OCA (−13.7%), which served as our positive control (Fig. 2H). Co-treatment of J2H-1702 and TGF- β1 in LX2 cells inhibited their expression of α -SMA, p-SMAD3, and 11βHSD1 (Fig. 2I). These results demonstrate that the pharmacological and genetic abrogation of 11βHSD1 induces depolarization of HSCs in a fibrotic microenvironment.

### The pharmacologic 11βHSD1 inhibitor J2H-1702 regulates pro-fibrotic genes in HSCs

Because our phenotypic results showed that J2H-1702 could control HSC activation, we next analyzed the effects of J2H-1702 on TGF-β1-induced HSC activation by detecting genome-wide alterations in gene expression in LX2 cells using RNA-sequencing (RNA-seq), a powerful approach for investigating drug-induced changes in this context.

**Fig. 3:**
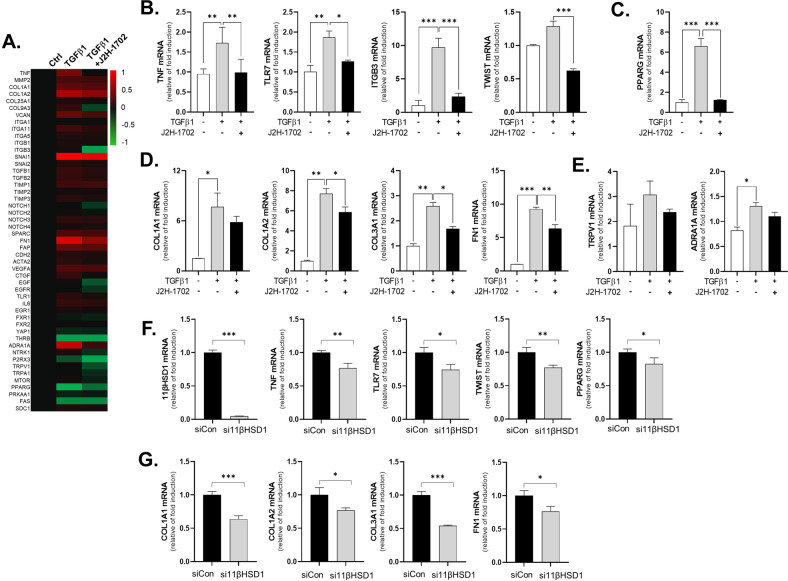
J2H-1702, an inhibitor of 11βHSD1, regulates pro-fibrotic genes in HSCs. **A** RNA-seq analysis of LX2 cells treated with 20 ng/ml TGFβ1 alone or with J2H-1702 was performed. mRNA expression of TNF, TLR7, ITGB3, TWIST **B**, PPARG **C**, COL1A1, COL1A2, COL3A1, Fibronectin (FN) **D**, TRPV1, and ADRA1A **E** were analyzed in LX2 cells and compared to control (20 ng/ml TGFβ-1 alone) and co-treatment with TGF-β1 and 1 µM J2H-1702. The data are shown as the mean ± standard deviation (SD) (*n* = 3). mRNA expression of 11βHSD1, TNF, TLR7, TWIST, PPARG **F**, COL1A1, COL1A2, COL3A1, and FN **G** were analyzed in treatment of 20 ng/ml TGFβ-1 in non-specific siRNA (siCon) or 11βHSD1 siRNA (si11βHSD1)-transfected LX2 cells. The data are shown as the mean ± standard deviation (SD) (*n* = 3). ^*^P < 0.05, ^**^P < 0.01 and ^***^P < 0.001 compared to the control group.

RNA-seq analysis showed that the expression of EMT-related genes was higher in TGF-β1-treated cells than in non-treated cells. Specifically, J2H-1702 downregulated collagen biosynthesis, growth factor signaling and ECM-receptor interaction, lipid storage, and fibromyalgia-related genes in TGF-β1-treated LX2 cells (Fig. 3A). Numerous autocrine or paracrine signaling molecules can induce liver fibrosis, so we next used RT-PCR to analyze the altered expression of genes selected as significant based on RNA-seq. We found that expression of TNF, TLR7, ITGB3, and TWIST, all of which are involved in NF-κB signaling (Fig. 3B), and PPARG, a key transcription factor associated with adipogenesis, were markedly decreased by treatment with J2H-1702 in TGF-β1-treated LX2 cells (Fig. 3C).

Because activated HSCs can accumulate ECM proteins, we investigated the effects of J2H-1702 on this accumulation of collagens (collagen I, III) and non-collagenous ECM protein (fibronectin, FN). Treatment of J2H-1702 significantly diminished levels of COL1A1, COL1A2, COL3A1, and FN in TGF-β1-treated LX2 cells (Fig. 3D).

Although RNA-seq showed that TRPV1 was the most highly upregulated gene after J2H-1702 treatment of TGF-β1-induced LX2 cells, we saw no significant change in the expression of TRPV1 and ADRA1A, both of which are fibromyalgia-related genes, following J2H-1702 treatment in TGF-β1-treated LX2 cells (Fig. 3E). Inhibition of 11βHSD1 expression by si11βHSD1 also attenuated expression of TNF, TLR7, TWIST, PPARG (Fig. 3F) and accumulation of ECM proteins (Fig. 3G) in TGF-β1-treated LX2 cells. Treatment of J2H-1702 and inhibition of 11βHSD1 expression showed similar mechanism in order to control of activation of hepatic stellate cells in fibrotic environment.

### J2H-1702 alleviated steatosis and fibrosis in MCD diet-fed mice

To analyze the effect of J2H-1702 on NASH, we employed an MCD diet mouse model that efficiently induces TG accumulation, severe damage, and progressive fibrosis in the liver.

**Fig. 4:**
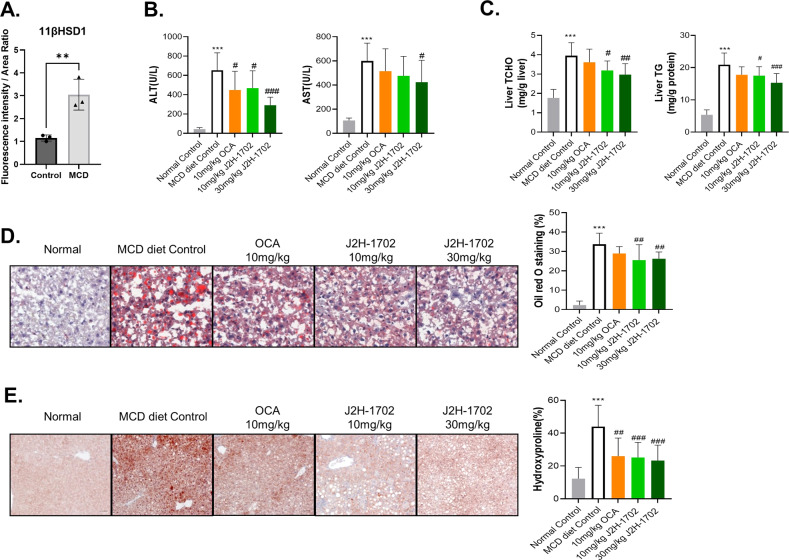
J2H-1702 ameliorates steatosis and fibrosis in MCD diet-induced NASH mice. Male C57BL/6 N mice were fed a normal chow diet (normal control) or an MCD diet to induce NASH and treated with 10 mg/kg OCA or 10 or 30 mg/kg J2H-1702. **A** Level of 11βHSD1 were increased in MCD-diet mouse livers in IHC staining. **B**, **C** ALT and AST levels were measured from collected plasma **B** and liver TC and TG levels were measured from hepatic tissues extracted at autopsy. **D**, **E** Oil Red O staining **D** and hydroxyproline staining **E** were performed, from which steatosis and fibrosis areas were measured. Values are expressed as mean ± SD (*n* = 10). ^***^P < 0.001 compared to the normal control group, ^#^*P* < 0.05, ^##^*P* < 0.01 and ^###^*P* < 0.001 compared to the MCD-diet vehicle control group.

First, we performed IHC of mouse liver tissues from normal chow diet and paired MCD diet NASH model and observed elevated 11βHSD1 expression in NASH mouse liver (Fig. 4A).

Serum concentrations of ALT and AST, enzymes indicating liver injury status, were increased significantly in MCD diet-fed mice. However, the J2H-1702-treated group had significantly decreased ALT and AST levels compared with the NASH group, suggesting that NASH-induced livers were somewhat protected when treated with J2H-1702 (Fig. 4B). MCD diet-fed mice exhibited remarkably reduced levels of serum TC and TG, whereas both parameters were significantly increased in hepatic tissues extracted at autopsy. In contrast, levels of hepatic TC and TG were reduced in J2H-1702-treated mice in a dose-dependent manner (Fig. 4C). Hepatic lipid deposition is another key component of NASH. Staining with Oil Red O, a histological marker for TG accumulation in hepatocytes, indicated that the extent of TG accumulation and macrovesicular steatosis was improved significantly in J2H-1702-treated mice in a dose-dependent manner (Fig. 4D). Quantification of hydroxyproline, a major constituent of collagen, is the most common method for evaluating tissue fibrosis/collagen deposition. Levels of hydroxyproline were also reduced in J2H-1702-mice groups dose-dependently (Fig. 4E). Altogether, these results showed that J2H-1702 treatment significantly improved hepatic steatosis and fibrosis in MCD- diet mice.

### J2H-1702 relieved steatosis and fibrosis in AMLN-fed mice

**Fig. 5:**
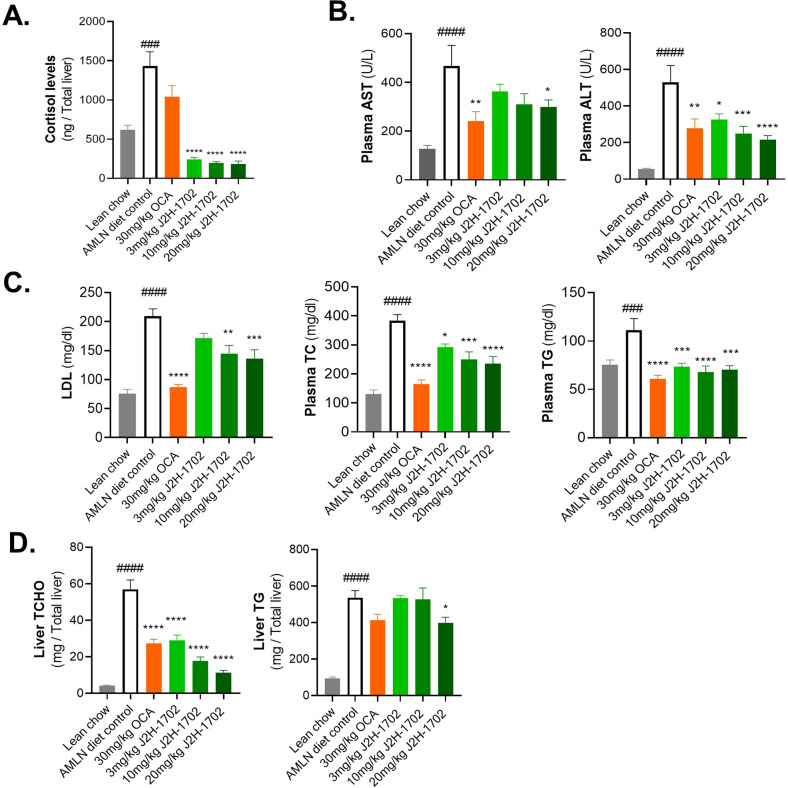
J2H-1702 ameliorates steatosis and fibrosis in AMLN diet-induced NASH mice. Male C57BL/6 N mice were maintained on an AMLN diet for 30 weeks and continued on the same diet during 12-week oral treatment with 30 mg/kg OCA or 3, 10, or 20 mg/kg J2H-1702. **A** Cortisol levels of liver tissues were estimated. **B**, **C** AST and ALT **B**, LDL, TC, and TG **C** levels were measured from collected mice plasma. **D** Liver TC and liver TG levels were measured from hepatic tissues obtained at autopsy. Values are expressed as mean ± SEM (*n* = 10). ^###^*P* < 0.001, ^####^*P* < 0.0001 vs Lean chow, ^*^P < 0.05, ^**^P < 0.01, ^***^P < 0.001 and ^****^P < 0.0001 vs. AMLN diet-fed control. One-way ANOVA followed by Dunnett’s test.

Although the MCD diet is widely used to induce NASH in mice, this model has limited value as a model for human NASH, as these mice exhibit reduced serum TC and TG levels, which differs from those in human patients with NASH. As a physiologically relevant dietary model for human NASH, we further employed the ALMN diet model to validate the efficacy of J2H-1702 efficacy against NASH. As expected, AMLN diet-fed mice exhibited significantly (131%, *p* < 0.001) increased liver cortisol level than the Normal chow-fed control. Treatment with J2H-1702 significantly decreased cortisol levels by fulfilling the original function of J2H-1702 in liver tissue, whereas OCA slightly diminished liver cortisol levels without statistical significance (Fig. 5A). In line with the results from the MCD diet-fed model, increased plasma AST and ALT levels in AMLN diet-fed mice were significantly reversed by treatment of J2H-1702 (Fig. 5B).

Regarding steatosis, AMLN diet controls displayed remarkably increased serum LDL (by 177%), TC (by 193%), and TG (by 47%) levels, contrary to those in MCD diet-fed mice. Oral administration of J2H-1702 significantly abated increases of LDL, TC, and TG levels in AMLN diet-fed mice (Fig. 5C). Intriguingly, AMLN diet-fed control mice showed significantly increased TC (by 1238%) and TG (by 475%) in hepatic tissues, and administration of J2H-1702 (3, 10, and 20 mg/kg) reduced hepatic TC more effectively than 30 mg/kg OCA. J2H-1702 (20 mg/kg) group resulted in decreased hepatic TC compared to AMLN diet-fed control mice group (Fig. 5D).

**Fig. 6:**
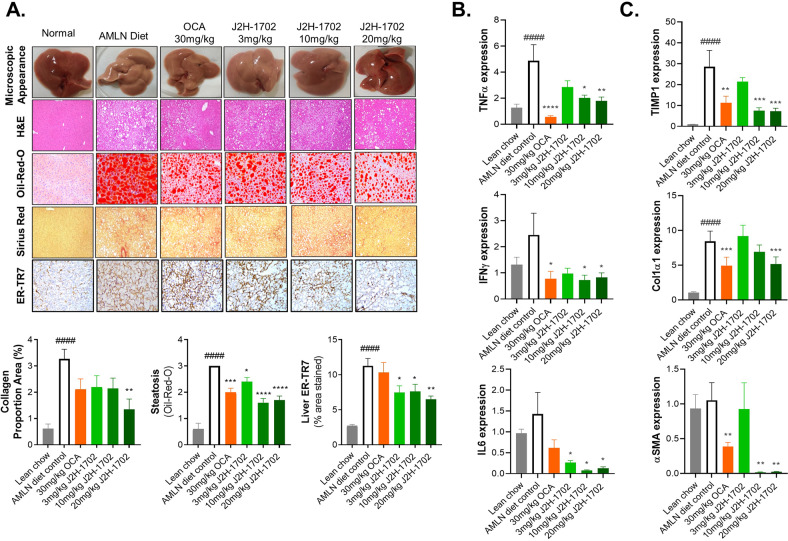
Histologic analysis and gene expression validates improvement of steatosis and fibrosis effects of J2H-1702 in Amylin liver NASH mouse model. Male C57BL/6 N mice were maintained on AMLN-diet for 30 weeks and were continued on the same diet during the treatment. Reference compound (30 mg/kg Obeticholic acid, OCA) and test compounds (3, 10, 20 mg/kg J2H-1702) were treated orally for 12 weeks. **A** Liver tissues of all the group animals were collected and were stained with H&E, Oil Red O, Sirius RED and ER-TR7. Collagen proportion area, steatosis, liver ER-TR7 levels were measured from tissue staining. **B**, **C** Gene expression of TNFα, IFNγ, IL6 **B** and TIMP1, Col1α1, α-SMA **C** were analyzed by quantitative RT-PCR in liver tissue. Values are expressed as Mean ± SEM. ^####^*P* < 0.0001 vs Lean chow, ^*^P < 0.05, ^**^P < 0.01, ^***^P < 0.001 and ^****^P < 0.0001 *vs* AMLN diet control. One way ANOVA followed by Dunnett’s test.

We also conducted histologic analysis to validate the efficacy of J2H-1702 on liver steatosis and fibrosis (Fig. 6A). AMLN diet-fed mice showed significantly increased (398%) collagen deposition compared with normal controls. In contrast with the vehicle control, 20 mg/kg J2H-1702 led to significantly decreased hepatic collagen deposits relative to OCA treatment. Oil Red O staining of liver tissues demonstrated that 10 mg/kg J2H-1702 treatment led to a 47% reduction in liver lipid accumulation, while 30 mg/kg OCA treatment caused a 33% lipid accumulation reduction in AMLN diet control mice. The ER-TR7 antibody detects an antigen present in and produced by reticular fibroblasts and can be utilized as a fibroblast marker. AMLN diet-fed control animals showed significantly increased ER-TR7 area (% stained) compared with the normal control, which significantly reduced by J2H-1702, more so than by OCA, in AMLN diet-fed mice (Fig. 6A).

Next, we analyzed mRNA expression levels of the inflammatory cytokines tumor necrosis factor-α (TNF-α), interferon-γ (IFN-γ), and interleukin-6 (IL-6) (Fig. 6B), as well as the fibrosis markers TIMP metallopeptidase inhibitor 1 (TIMP1), collagen type 1 alpha 1 (Col1A1), and α-SMA, in liver tissue from AMLN diet-fed mice (Fig. 6C). Gene expression data reveal that treatment with J2H-1702 significant reduced liver TNF-α, IFN-γ, IL-6, TIMP-1, Col1α1, and α-SMA mRNA expression levels compared to AMLN diet-fed control animals.

Altogether, these results suggest that J2H-1702 treatment reverses hepatic steatosis and fibrosis through inhibition of NASH-induced gene expression in AMLN diet-fed mice.

### J2H-1702 abated inflammation and fibrosis in toxicity-induced NASH mice

**Fig. 7:**
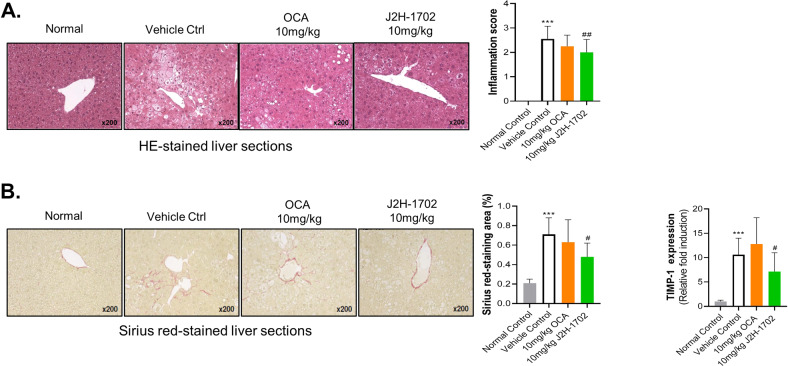
J2H-1702 reduces inflammation and fibrosis in chemically-induced NASH STAM mouse model. Male C57BL/6 J mice were tested with streptozotocin injection and high fat diet (HFD) feeding to induce NASH model. HFD were starting at 4 week and administration of compounds (10 mg/kg OCA for reference, 10 mg/kg J2H-1702 for test, orally) was maintained for 6 weeks. **A** Histopathology was performed by H&E staining and inflammation score was measured in liver tissues. **B** Sirius red-staining was examined to measure fibrosis area. TIMP gene expression was analyzed by quantitative RT-PCR. Values are expressed as Mean ± SD (*n* = 10). ^***^P < 0.001 compared to the normal control group, ^#^*P* < 0.05 and ^##^*P* < 0.01 compared to the STAM vehicle control.

We also administered J2H-1702 in the stelic animal model (STAM) mice with toxicity-induced NASH using STZ and a HFD. This NASH model is widely used primarily because it recapitulates the full spectrum of liver disease from NAFLD to HCC. H&E-stained liver sections from the vehicle group exhibited micro- and macrovesicular fat deposition, hepatocellular ballooning, and inflammatory cell infiltration compared with the normal control group. Treatment with J2H-1702 led to a significant decrease in inflammation score compared with OCA (Fig. 7A). J2H-1702 also caused a significant reduction of the pathological deposition of collagen as demonstrated by Sirius red staining, as well as decreased TIMP-1 mRNA expression in the livers of STAM mice (Fig. 7B). These results showed that J2H-1702 exerts anti-inflammatory and anti-fibrosis effects in the NASH STAM model.

## Discussion

Because NASH is a multifactorial disease, various targets have been investigated when developing approaches for screening small molecules to identify and evaluate drugs that may treat hepatic fibrosis. For example, OCA (FXR agonist), aramchol (SCD1 inhibitor), cenicriviroc (CCR2/CCR5 antagonist), elafibranor (pan-PPAR agonist), and resmetirom (THR- β agonist) are being evaluated in phase 3 trials [2]. Although these five pharmacologic agents have been deemed effective against liver fibrosis, there is still a major unmet need for drugs that can resolve NASH and reverse liver fibrosis; moreover, no validated target exists for novel anti-fibrotic compounds [18, 19].

In this study, we applied 3D cultures in drug discovery and repositioning to find valuable targets and drugs for efficient anti-fibrotic therapy. Our previous work suggested that J2H-1702, a novel inhibitor of 11βHSD1, has great potential as a novel candidate drug for treating diabetes and metabolic diseases, with pharmacokinetic and safety profiles [15, 16]. In Phase I clinical trial, J2H-1702 was shown to be well-tolerated with mild adverse events after oral administration in healthy male subjects, not requiring any intervention or treatments. According to single ascending dose (SAD) and multiple ascending dose (MAD) studies, dose-dependent exposure in plasma was confirmed compatible with once-daily dosing within therapeutic range. Here, we applied J2H-1702 in hepatic fibrosis drug discovery studies of MCHS models, which mimic a fibrotic and immune microenvironment, to find alternative/added indications using MCHS models. To analyze on which MCHS cell types J2H-1702 acts, we evaluated expression of 11βHSD1, a regulator of glucocorticoids, in parenchymal and non-parenchymal cells in the liver. Notably, J2H-1702 mainly targets the control cortisol production and EMT activation of HSCs, rather than hepatocytes, in the fibrotic environment (Fig. 1).

Because activation of HSCs into myofibroblasts is the central driver of hepatic fibrosis, reversal of HSC activation is emerging as a new strategy for developing novel therapies for liver disease [20–22]. Various signaling pathways and intracellular events have been suggested as contributors to HSC activation, such as fibrogenic cytokines and growth factors, innate immune signaling, hedgehog signaling, nuclear receptors, GPCRs, and metabolic alterations, among others [23]. Recently, 11βHSD1 was reported to play an important role in obesity and metabolic diseases, as well as in HSC activation, and as a result, has become a target of interest for treating liver fibrosis [13, 14, 24, 25]. In the current study, we found that TGF-β1-mediated fibrogenic morphology and signaling in HSCs were significantly abrogated by pharmacological inhibition and genetic suppression of 11βHSD1 (Fig. 2).

Regarding the mechanism of action of J2H-1702 during liver fibrosis, TNF showed the greatest expression change following J2H-1702 administration in TGF-β1-treated LX2 cells according to RNA-seq analysis. Specifically, mRNA expression of TNF was significantly inhibited by J2H-1702 in these cells and in AMLN diet-fed mice. TNFα is a key player involved in HSC survival [26, 27], hepatocyte death, and immune cell activation, all of which are associated with advanced liver fibrosis [28]. Although TNF upregulates 11βHSD1 expression in inflammatory diseases [29–31], we found that inhibition of 11βHSD1 can control TNF expression during liver fibrogenesis. As TNF mainly acts through NF-κB signaling to regulate downstream gene pathways, we also observed that expression of genes related to NF-κB activation (TLR7, ITGB3, and TWIST), which generally promote inflammation, fibrosis, and hepatocarcinogenesis in non-parenchymal cells [32–34], were downregulated after treatment of J2H-1702 (Fig. 3). Collagens are the most abundant structural ECM protein in the liver, and disproportionate levels of collagen result in altered cellular phenotypes and architectural distortion with abnormal blood flow. Importantly, 11βHSD1 inhibition effectively diminished levels of collagens in TGF-β1-treated LX2 cells and in NASH mouse models.

In contrast to our results, the Zoi Michailidou group reported that depletion of 11βHSD1 expression exacerbates hepatic myofibroblast activation and liver fibrosis in a CCl_4_-induced liver injury model in 11βHSD1-deficient mice, but they did not exclude possibility that efficacy may vary depending when the 11βHSD1 inhibitor is given in the liver injury and repair process [14]. To verify the efficacy of 11βHSD1 inhibitors for NASH treatment, we applied J2H-1702 to various diet (MCD, AMLN)—and toxicity (STAM model)-induced NASH mice. Administration of J2H-1702 reduced steatosis and fibrosis in diet-induced NASH mice and attenuated inflammation and fibrosis in the toxicity-induced NASH mice. Collectively, our results show that J2H-1702 could alleviate liver fibrosis with a concomitant decrease of ALT and AST in NASH mice (Table 1).

**Table 1:** J2H-1702 ameliorates liver fibrosis with decreasing of ALT and AST in NASH mice models.

Disease model	MCD	STAM	AMLN
Steatosis	Reduction	N/C	Reduction
Inflammation	N/C	Reduction	N/C
Fibrosis	Reduction	Reduction	Reduction

*N/C No significant changes

Because the mechanisms of 11βHSD1 is complex and its role during NASH progression is still controversial, targeting 11βHSD1 should be carefully considered as a new therapeutic approach for the liver fibrosis.

## Materials and methods

### Chemical agents

TGF-β1 (100–21 C; 10 μg) was purchased from Peprotech. Phorbol 12-myristate 13-acetate (PMA; P8139–1MG) was purchased from Sigma. Obeticholic acid (OCA) and J2H-1702 were synthesized by J2H Biotech Inc.

### Cell lines and culture

Huh7, Hep3B, SNU475, and SNU449 cells were obtained from the Korean Cell Line Bank. Human umbilical vein endothelial cells (HUVECs) were obtained from Promocell, Fa2N-4 cells were purchased from XenoTech, and LX2 cells were obtained from Millipore. WI38 and THP-1 cells were purchased from the American Type Culture Collection (Manassas). All cells were maintained at 37 °C in a humidified atmosphere of 5% CO_2_. Huh7 cells were cultured in “complete medium” composed of RPMI-1640 medium (Welgene) supplemented with 10% fetal bovine serum (FBS; Gibco), 1× penicillin-streptomycin (P/S; Gibco). LX2 cells were cultured in Dulbecco’s Modified Eagle medium (Welgene) supplemented with 2% FBS and 1× P/S. For HUVECs, endothelial basal medium was purchased from Promocell. All reagents and siRNA are provided in Supplementary Information Table 1.

### Generation of 3D spheroids and MCHSs

HCC cells (Huh7) and LX2 cells were co-cultured at a ratio of 1:1 or 7:3 in one well. In addition, to generate only 3D spheroids, Huh7 or Fa2N-4 cells were seeded at a density of 6 × 10^3^ cells/well in 96-well round-bottomed, ultra-low attachment microplates (Corning B.V. Life Sciences). The cells were incubated for 3 days at 37 °C in a humidified atmosphere of 5% CO_2_. Huh7 cells were seeded with stromal cells (LX2 cells, WI38 cells, and HUVECs) to generate MCHSs in the same manner.

### Cortisol assay

LX2 cells, HUVECs, and THP-1 cells were seeded at a total density of 4 × 10^5^ cells/well in a 60-mm dish (Falcon) at 37 °C for 24 h. THP-1 cells were treated with 50 ng/ml PMA to differentiate macrophages. MHCSs were seeded at a density of 6 × 10^3^ cells/well in 96-well round-bottomed, ultra-low attachment microplates (Corning B.V. Life Sciences) at 37 °C for 72 h. The cells (MCHS, LX2, THP-1, and HUVECs) were incubated in complete medium containing 1 μM cortisone for 30 min, and then 0 or 20 ng/ml TGF-β1 and 0, 0.5, 1, or 2 μM J2H-1702 were added. After 2 days, the conditioned medium was centrifuged at 12,000 × *g* for 10 min and the supernatant was collected. To measure cortisol levels in the supernatant, the Cortisol Parameter Assay Kit (R&D Systems) was used according to the manufacturer’s instructions.

### Immunofluorescence (IF) assay

For immunofluorescence assay of 11βHSD1 in spheroid, the spheroids were fixed in 4% paraformaldehyde (PFA) and washed in DPBS with Tween 20 (DPBS-T). After than the samples were permeable incubated using 0.3% Triton X-100 in 10 min, and blocked in 10% normal goat serum (NGS) in 1 h at room temperature. The samples were immunoblotted with primary antibodies (11βHSD1 and F-actin) and secondary fluorophore-conjugated antibodies. In IF staining of liver patients samples, the patient samples were purchased AccuMax™ tissue microarray (ISU ABXIS, Seoul, Korea). The slides were deparaffinized in xylene, and hydrated within 100–50% alcohol gradient step by step. After washing 2 times with DPBS-T for 15 min, the samples were warmed using microwave with citrate buffer. After cooling, the slides were washed 3 times in TBS-T for 5 min each, and immunoblotted with primary antibody of 11βHSD1 in 3% bovine serum albumin (BSA) overnight at 4 °C. After immunoblotting, the secondary fluorescence conjugated antibodies and Cell nuclei stained using Hoechst 33342 (1:1000, MOP-H3570; ThermoFisher). After washing the samples were mounted, and detected using automated high-content imaging system (OPERETTA, PerkinElmer) and confocal laser scanning microscope (CLSM II; LSM710A, Carl Zeiss). The images were analyzed by an in-house software tool and HARMONY 3.5.1. (PerkinElmer).

### Western blot

The cells were lysed using extraction buffer (Thermo Scientific) and measured for protein concentration with a BCA protein assay kit (Pierce). The cell lysates were separated by 8–15% SDS-PAGE and transferred to a nitrocellulose (NC) membrane (Pall Corporation). Membranes were blocked with 5% skim milk in Tris-buffered saline/Tween 20 (TBS-T) buffer for 1 h at RT. After washing steps with TBS-T buffer, NC membranes were incubated with rabbit monoclonal anti-α-SMA (E184, 1:1000), rabbit polyclonal anti-HSD11B1 (1:500), rabbit polyclonal anti-N Cadherin (1:1000), mouse monoclonal anti-Vimentin (RV202, 1:1000) (all from Abcam), rabbit monoclonal anti-phospho-Smad3 (Ser423/425) (C25A9, 1:500), rabbit monoclonal anti-SMAD2/3 (1:1000), rabbit monoclonal anti-Snail (C15D3, 1:1000) (all from Cell Signaling Technology), rabbit polyclonal anti-Collagen I (Novus Biologicals, 1:500), and mouse monoclonal anti-β-actin (Sigma-Aldrich, 1:3000) for 16 h at 4 °C. After washing step, with TBS-T buffer, the membranes were incubated with horseradish peroxidase-conjugated secondary antibody (Cell Signaling Technology, 1:5000), and the specific bands were visualized by enhanced chemiluminescence (ECL; Thermo Scientific).

### Quantitative real-time PCR

RT-PCR was performed using an iScript cDNA Synthesis Kit (Bio-Rad). The information of target primers is attached in Supplementary Table 1. Real-time PCR mixtures containing SYBR Green (Applied Biosystems) were processed in the StepOnePlus real-time PCR system (Applied Biosystems) as follows. The threshold cycle (CT) was defined as the fractional cycle number at which the fluorescence exceeds the fixed threshold. CT values were normalized to those of GAPDH or 36B4 and calculated according to the mathematical model R = 2^−ΔΔCT^ method. All real-time PCR was performed in triplicate, and the data were presented as the Mean ± standard deviation (SD).

### Animal experiment

Male C57BL/6 N mice 5–6 weeks of age were fed a methionine/choline-deficient (MCD) diet, which started at week 6 and was maintained for 10 weeks. From week 16, the reference compound (OCA) or test compound (J2H-1702) was given orally with the continued MCD diet. For the AMLN model, male C57BL/6 N mice 5–6 weeks of age were maintained on an Amylin liver NASH (AMLN) diet for 30 weeks to induce NASH for 30 weeks, which continued during treatment with OCA or J2H-1702 for 12 weeks. This diet consisted of 40 kcal% fat (mostly palm oil), 20 kcal% fructose and 2% cholesterol provided ad libitum. All diet-induced NASH mice were orally administered diet-control (MCD diet control or AMLN diet control), OCA, or J2H-1702 daily before the start of a dark cycle from day 0 to 84 days. Animals were then fasted for 4 h on the terminal day, dosed with the test or reference compound 1 h prior to blood collection, and then sacrificed. NASH was induced in STAM mice by a single subcutaneous injection of 200 μg streptozotocin (STZ, Sigma-Aldrich) solution 2 days after birth and feeding with a high-fat diet (HFD: 57 kcal% fat) after 4 weeks of age. Test compound (J2H-1702) or OCA was administered orally from 6 weeks to 12 weeks. STAM mice were sacrificed and blood and tissues were collected at 12 weeks. J2H-1702 (10 or 30 mg/kg for MCD mice; 3, 10, or 20 mg/kg for AMLN mice; 10 mg/kg for STAM mice) and OCA (10 mg/kg for MCD and STAM mice; 30 mg/kg OCA for AMLN mice) were formulated in 1% (v/v) Tween 80 and 99% (v/v) of 0.5% methyl cellulose and diluted with vehicle. Dosing volumes were calculated based on body weight taken on the day of dosing. Plasma alanine aminotransferase (ALT), aspartate aminotransferase (AST), triglycerides (TG), total cholesterol (TC), glucose, and low-density lipoprotein levels were measured from collected plasma. TG, TC, hydroxyproline, and cortisol estimations were performed in liver tissues. Histopathological examination and staining with hematoxylin & eosin (H&E), Sirius Red, and Oil Red O were performed for collected liver tissues. Immunohistochemistry (IHC) using the fibroblast marker ER-TR7 and gene expression were analyzed in collected liver tissues. All animal experiments were received and approved (Approval No: B-047 and 18-KE-042) by the institutional animal ethics committee (IAEC) and KNOTUS IACUC.

### Statistical analysis

All experiments were performed at least three times. The data are shown as the mean ± SD or mean ± standard error of the mean (SEM). A Student’s t-test or One way ANOVA followed by Dunnett’s test were used to assess statistically significant differences using Microsoft Excel or GraphPad Prism. No statistical methods were used to predetermine the sample size. Mice were randomly allocated to experimental groups. No blinding was performed in any of the experiment. The variances were statistically similar between the compared groups.

## Supplementary information

Supplementary Information Table 1

Original WB blots

Original Data File

Author information

Checklist

## Data Availability

The datasets generated and/or analysed during the current study are available from the corresponding author on reasonable request.
