# Supplementary material for: Inhibition of 11β-hydroxysteroid dehydrogenase 1 relieves fibrosis through depolarizing of hepatic stellate cell in NASH
Source: Cell Death Dis. 2022 Nov 29;13(11):1011. doi: 10.1038/s41419-022-05452-x (PMC9709168; doi:10.1038/s41419-022-05452-x)
Supplement: Supplementary file 1 — Supplementary Information Table 1 [file 41419_2022_5452_MOESM1_ESM.docx]

**Supplementary Information**

**Supplementary Information Table 1. Primer sequences for qRT-PCR**

| **GENE** | **SEQUENCE (5' --> 3')** | | |
| --- | --- | --- | --- |
| TNF | forward |  | CTCGAACCCCGAGTGACAAG |
|  | reverse |  | TATCTCTCAGCTCCACGCCA |
| TLR7 | forward |  | CACATACCAGACATCTCCCCA |
|  | reverse |  | CCCAGTGGAATAGGTACACAGTT |
| TRPV1 | forward |  | CTGCCCGACCATCACAGTC |
|  | reverse |  | CTGCGATCATAGAGCCTGAGG |
| ADRA1A | forward |  | CGCTACCCAACCATCGTCAC |
|  | reverse |  | GAACAGGGGTCCAATGGATATG |
| COL1A1 | forward |  | CGCTGGTTTCGATTCAGCT |
|  | reverse |  | ACATTGGCATCATCAGCCCG |
| COL1A2 | forward |  | CTGGCCCCAATGGATTTGCT |
|  | reverse |  | CCGTTTTCACCCTTAGGCCC |
| COL3A1 | forward |  | GGTAGCCCTGGTGAGAGAGG |
|  | reverse |  | CGGAGCCCCTCTTTCTCCTT |
| FN1 | forward |  | AGCTTTGTGGTCTCCTGGGT |
|  | reverse |  | ACTGTGGCTCATCTCCCTCC |
| HSD11b1 | forward |  | TGGCTTATCATCTGGCGAAGA |
|  | reverse |  | AGGCAGTGGGATACCACCT |
| GAPDH | forward |  | GACGAACATGGGGGCATCAG |
|  | reverse |  | GCGTCACCACCATGGAG |
| ITGB3 | forward |  | CATGAAGGATGATCTGTGGAGC |
|  | reverse |  | AATCCGCAGGTTACTGGTGAG |
| PPARG | forward |  | ACCAAAGTGCAATCAAAGTGGA |
|  | reverse |  | ATGAGGGAGTTGGAAGGCTCT |
| TWIST | forward |  | GTCCGCAGTCTTACGAGGAG |
|  | reverse |  | GCTTGAGGGTCTGAATCTTGCT |
| TNFα | forward |  | CTCTTCTGCCTGCTGCACTTTG |
|  | reverse |  | ATGGGCTACAGGCTTGTCACTC |
| TIMP1 | forward |  | TGAGCCCTGCTCAGCAAAGA |
|  | reverse |  | GAGGACCTGATCCGTCCACAA |
| IFN-γ | forward |  | TCGGTAACTGACTTGAATGTCCA |
|  | reverse |  | TCGCTTCCCTGTTTTAGCTGC |
| IL6 | forward |  | ACTCACCTCTTCAGAACGAATTG |
|  | reverse |  | CCATCTTTGGAAGGTTCAGGTTG |
| α-SMA | forward |  | AAAAGACAGCTACGTGGGTGA |
|  | reverse |  | GCCATGTTCTATCGGGTACTTC |
| 36B4 | forward |  | TTCCAGGCTTTGGGCATCA |
|  | reverse |  | ATGTTCAGCATGTTCAGCAGTGTG |
