# Supplementary figures and images for: Inhibition of 11β-hydroxysteroid dehydrogenase 1 relieves fibrosis through depolarizing of hepatic stellate cell in NASH
Source: Cell Death Dis. 2022 Nov 29;13(11):1011. doi: 10.1038/s41419-022-05452-x (PMC9709168; doi:10.1038/s41419-022-05452-x)

**Fig 1A.**

Full and uncropped western blot for Figure 1A

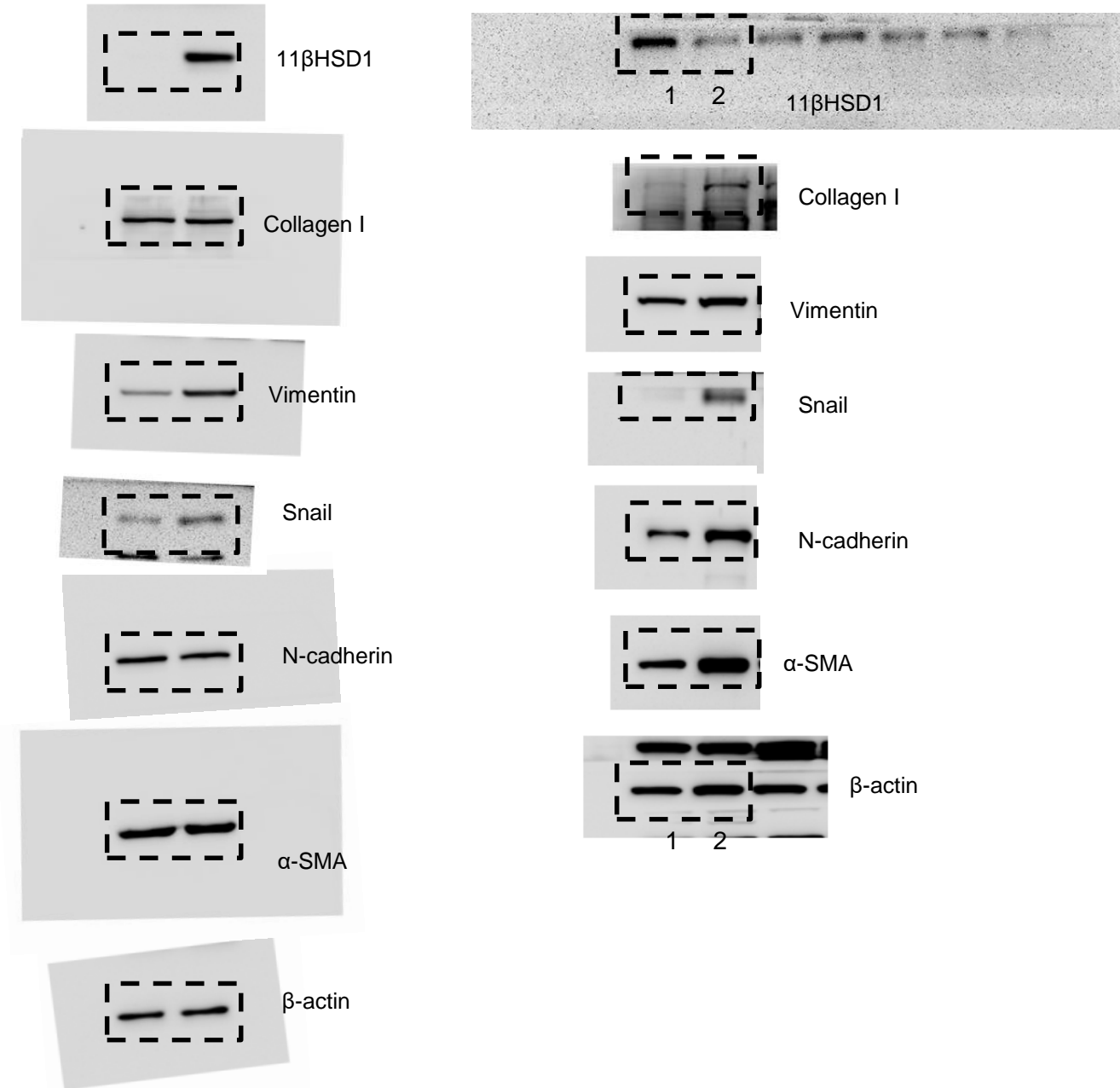

**Fig 1B.**

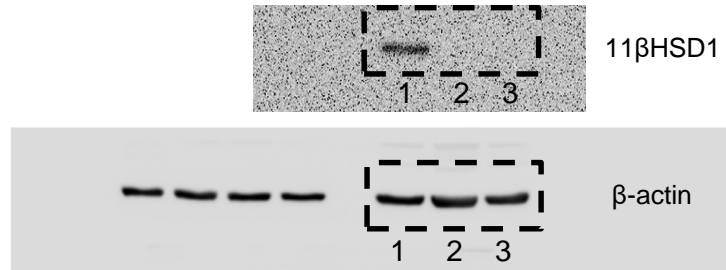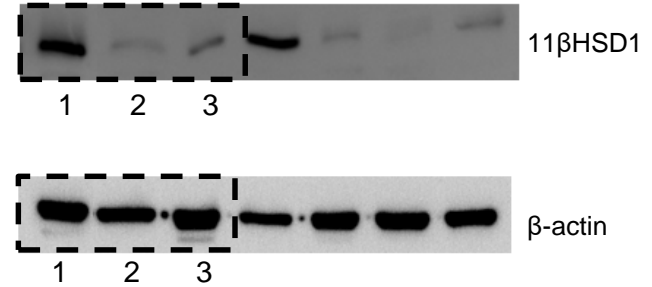

**Fig 1C.**

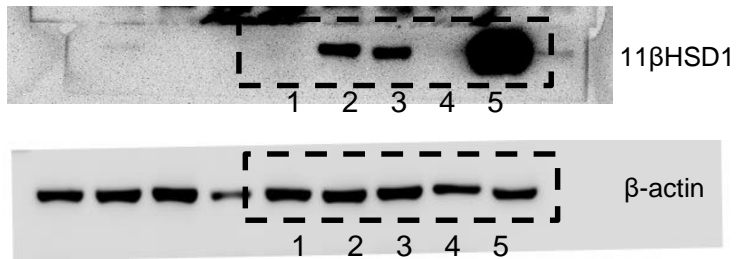

**Fig 1D.**

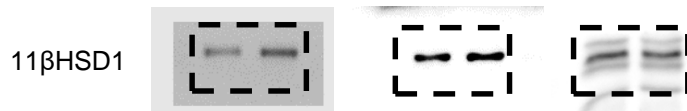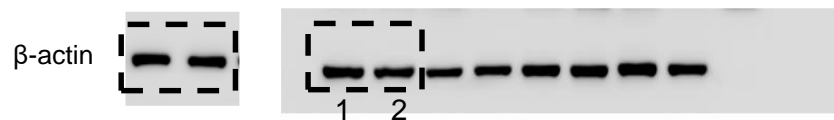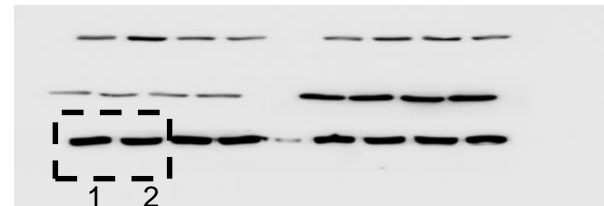

**Fig 1I.**

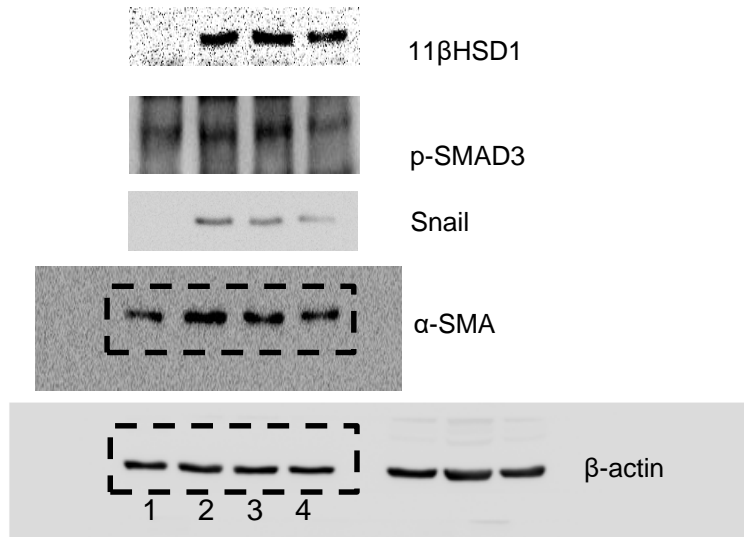

**Fig 2B.**

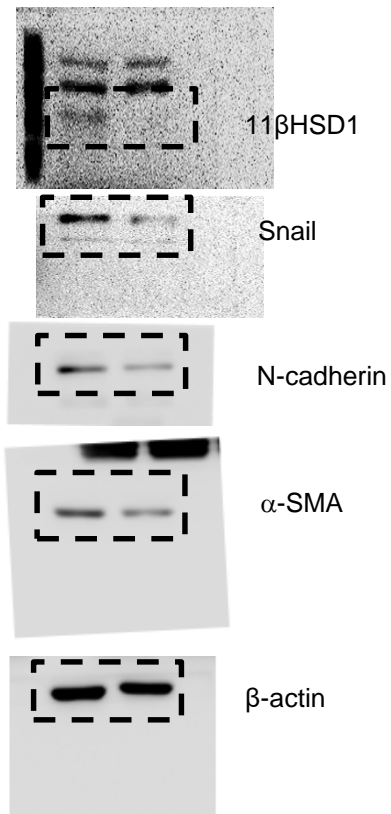

**Fig 2E.**

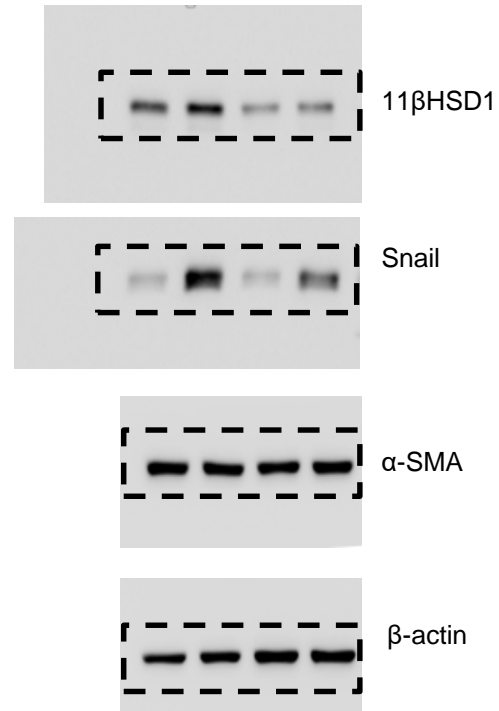

**Fig 2G.**

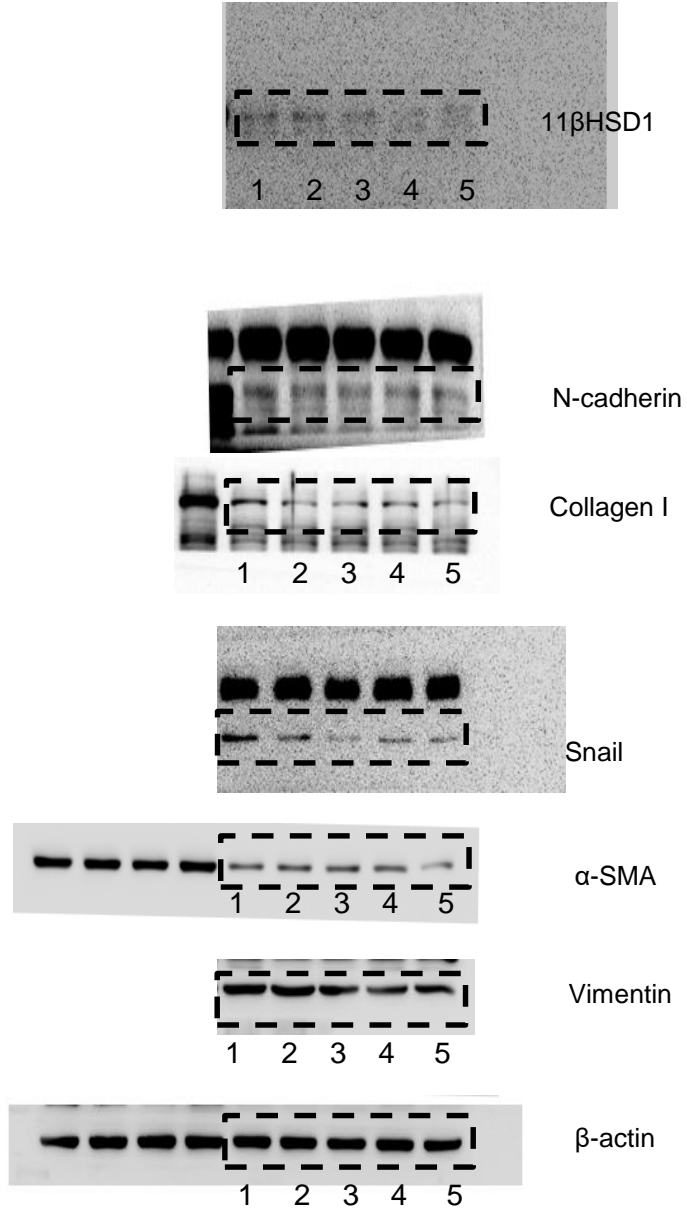

**Fig 2I.**

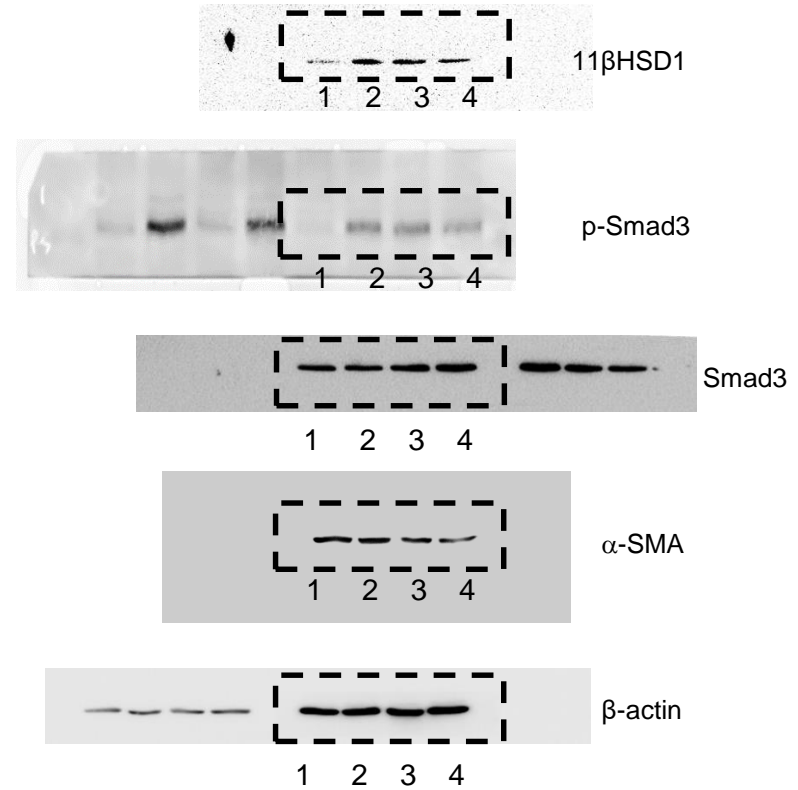

Supplement: Supplementary file 2 — Original WB blots [file 41419_2022_5452_MOESM2_ESM.pdf]
