# Supplementary material for: Inhibition of 11β-hydroxysteroid dehydrogenase 1 relieves fibrosis through depolarizing of hepatic stellate cell in NASH
Source: Cell Death Dis. 2022 Nov 29;13(11):1011. doi: 10.1038/s41419-022-05452-x (PMC9709168; doi:10.1038/s41419-022-05452-x)
Supplement: Supplementary file 4 — Author information [file 41419_2022_5452_MOESM4_ESM.docx]

**Author information**

Authors and Affiliations

**Advanced Biomedical Research Lab, Institut Pasteur Korea, 16, Daewangpangyo-ro 712 beon-gil, Bundang-gu, Seongnam-si, Gyeonggi-do, 13488, Republic of Korea**

Su-Yeon Lee, Sanghwa Kim, Yeonhwa Song, Namjeong Kim, Haeng Ran Seo

**Medicinal Chemistry, Institut Pasteur Korea, 16, Daewangpangyo-ro 712 beon-gil, Bundang-gu, Seongnam-si, Gyeonggi-do, 13488, Republic of Korea**

Inhee Choi

**R&D center, J2H Biotech Inc., Saneop-ro 156 beon-gil , Gwonseon-gu, Suwon-si, Gyeonggi-do, 16648, Republic of Korea**

Hyung Chul Ryu, Jee Woong Lim, Hyo Jin Kang, Jason Kim
